# Microalgae strains isolated from piggery wastewater in Ecuador: Effective nitrogen compound removal and growth potential in extremophile conditions

**DOI:** 10.1016/j.btre.2025.e00883

**Published:** 2025-02-10

**Authors:** Karla Flores-Zambrano, Wilson Tapia, Pablo Castillejo

**Affiliations:** a Grupo de Investigación en Biodiversidad, Medio Ambiente y Salud (BIOMAS), Universidad de Las Américas (UDLA), Quito, Ecuador; b Carrera de Ingeniería en Biotecnología, Facultad de Ingeniería y Ciencias Aplicadas, Universidad de las Américas (UDLA), Quito, Ecuador

**Keywords:** Microalgae barcodes, Pig effluent, Phycoremediation, Extremophile

## Abstract

Effluents generated by anthropogenic activities are a significant source of pollution and eutrophication in natural water bodies. In Ecuador, the increase in pig production has exacerbated this issue due to the untreated discharge of pig effluents. This study focused on the characterization of native microalgae present in pig effluents and the evaluation of their capacity to remove nitrogenous compounds under various conditions, with the aim of identifying efficient strains for phycoremediation. Four microalgal strains were isolated and molecularly identified as *Radiococcus polycoccus, Chlorolobion braunii, Micractinium* sp., and *Desmodesmus multivariabilis*. The cultures were exposed to initial concentrations of 100 mg L^-1^ N-NH₄ and 49.97 mg L^-1^ N-NO₃ for 12 days to assess their cellular growth and nutrient removal rates. Growth kinetics were analyzed under conditions of 2000 mg L^-1^ N-NH₄ and extreme pH levels of 3 and 10. *Chlorolobion braunii* demonstrated the highest productivity, achieving a removal of 67.73 % of N-NH₄ and 30.59 % of N-NO₃, and reached the highest cellular density under extreme ammonium conditions, being the only strain capable of growing at acidic pH. Conversely, *Micractinium* sp. exhibited the highest growth under alkaline conditions. These results highlight the promising potential of native microalgae from pig effluents for wastewater remediation and their adaptation to environmental conditions.

## Highlights

• Four strains isolated from pig wastewater removed more than 60 % of N-NH_4_ over a 12-day period.
• First report on nitrogen removal kinetics for *Radiococcus Polycoccus.*
• Indigenous *Chlorolobion braunii* survived and grew at pH 3 and showed high adaptability, thriving at 2000 mg L^-1^ N-NH_4_.
• Kinetic growth monitoring reveals the adaptability of *Chlorolobion Braunii* to extreme ammonium-nitrogen concentrations and acidic pH conditions.

## Graphical abstract

**Figure:**
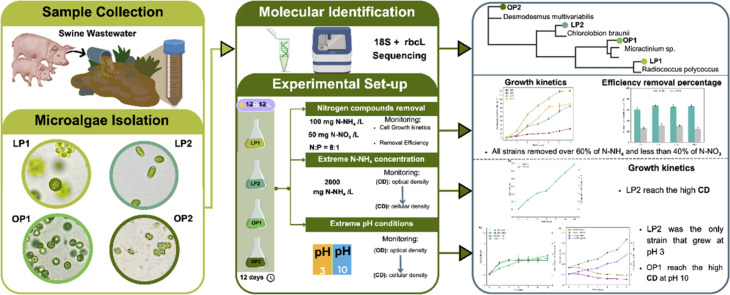

## 1 Introduction

The scarcity of water due to climate change, anthropogenic pollution, and population growth constitute a significant challenge globally. Porcine leachate is one of the pollutants that poses significant environmental challenges as it can contain a variety of pollutants that put at risk the ecosystem integrity: high concentrations of organic matter, nutrients (such as nitrogen and phosphorus), pathogens, antibiotics, and trace metals [1,2]. When these leachates infiltrate the soil or are carried off-site by surface runoff or groundwater flow, they can contaminate surface water bodies, groundwater reservoirs, and surrounding land [3]. On-site treatment technologies, such as anaerobic digestion, aerobic treatment, and constructed wetlands, can be employed to treat porcine leachates before they are discharged into the environment [4]. These treatment systems can remove or reduce contaminants in leachates, making them less harmful to ecosystems and human health.

Phycoremediation, a form of bioremediation utilizing algae, has emerged as a promising approach to address water pollution challenges [5,6]. By harnessing the natural ability of algae to absorb and metabolize pollutants, phycoremediation offers a sustainable and cost-effective solution for restoring water quality in contaminated environments [7]. Several microalgae species provide promising solutions for ammonium removal due to their efficient nutrient uptake mechanisms [8]. Among these species, some of the most utilized include the genus *Chlorella* [9], *Scenedesmus* and *Nannochloropsis* [10].

Some research showed that the utilization of indigenous microalgae from contaminated sources enables more effective nutrient removal processes than foreign inocula ​[11]​. These strains are naturally adapted to specific environments and climates, endowing them with greater competitiveness, efficiency, and sustainability in industrial applications [12]. Thus, the characterization of microalgae isolated from porcine leachates is essential for the selection of efficient strains that can thrive in swine wastewater with high levels of nitrogen compounds [13]. The collection, screening, and identification of local microalgae provide valuable information to address the existing knowledge gap and are fundamental requirements for the development of highly efficient phycoremediation systems in biodegradation [12].

Family-owned pig farms play a vital role in Ecuador's agricultural landscape, contributing significantly to the country's economy and food security, but also to the pollution of the environment. Hence, this study aimed to evaluate the potential of four microalgal strains isolated from piggery leachates to remove nitrogen compounds and assess their growth capacity under extreme conditions, including high ammonia concentrations and extreme pH levels.

## 2 Material and methods

### 2.1 Sample collection

Sampling was conducted following the guidelines of the Ecuadorian Technical Standard NTE INEN 2169:2013, which addresses water quality, sampling, and sample preservation. Samples were taken from the oxidation pool for piggery effluents of the "Bella María" estate and piggery wastewater from waterfall “La Paz” in Nanegalito, Quito, Ecuador. Samples (2 L) were collected from each sampling point in plastic containers [14]. For future physicochemical analyses, 500 mL of wastewater was collected in six sterilized dark glass containers. Samples were kept in a portable refrigerator until transported to the Environmental Sample Analysis Laboratory in the research area of the Universidad de las Américas (UDLA), Quito-Ecuador.

### 2.2 Microalgae isolation

Homogenized leachate (300 µL) was spread onto Bold's basal medium (BBM) agar plates (2 % w/v). After a single colony of microalgae appeared, strains were isolated through consecutive rounds of subculturing using the streak plate technique until achieving a pure culture. Validation was conducted using bright-field microscopy [15]. Unialgal cultures were then scaled up in liquid BBM to 500 mL as a stock inoculum required for the experimental setup. Additionally, an aliquot (10 mL) was stored long-term in 10% glycerol-BBM at −80 °C [16].

### 2.3 Molecular identification

DNA was extracted from the colonies of each isolated strain following [17], with an additional step of washing the colonies with Type 1 water and a prior step of mechanical disruption by spinning a sterile inoculation needle against the inner surface of a 1.5 mL tube to vigorously homogenize the pellet. Two molecular markers were utilized: the 18S rDNA and rbcL. The primer pair targeting the 18S rDNA V1-V3 region was employed to amplify a 563 bp fragment, as described by [18]. Two regions of the rbcL gene, spanning from positions 192–657 and 375–1089, respectively, were amplified using primers as described by [19]. Amplification was performed with 2X GoTaq® Green Master Mix (Promega), with 20 to 30 ng of extracted DNA. The reaction consisted of an initial denaturation step at 95 °C for 2 min, followed by 35 cycles as follows: 45 s at 95 °C, 30 s annealing at the proper temperature for each primer pair, 1 min at 72 °C, and a final extension step at 72 °C for 5 min.

Direct Sanger sequencing was performed on an ABI 3500xL genetic analyzer (Applied Biosystems, Waltham, MA, USA) using a BigDye Terminator v3.1 sequencing kit. Sequences were edited with MEGA X software [20] and compared against the GenBank database at the National Center for Biotechnology Information (NCBI) using the Basic Local Alignment Search Tool (BLAST). Reference sequences were retrieved from the GenBank database and aligned them with barcoding sequences obtained from our strains using the Muscle Algorithm [21]. The application of the General Time Reversible model, incorporating a discrete *G* + *I* distribution, was guided by the corrected Akaike Information Criterion (AICc) within MEGAX's ML model selection feature. Tree topologies and branch lengths for both markers were jointly computed utilizing the maximum-likelihood method (ML) in PhyML 3.0 [22].

### 2.4 Experimental set-up and culture conditions

#### 2.4.1 Synthetic culture medium

The synthetic medium used in all experiments was a modified standard APP medium [23,24] with the following base composition (mg L⁻¹): 12 MgCl₂·6H₂O, 15 MgSO₄·7H₂O, 0.08 FeCl₃·6H₂O, 0.1 Na₂EDTA·2H₂O, 0.185 H₃BO₃, 0.415 MnCl₂·4H₂O, 0.003 ZnCl₂, 0.0015 CoCl₂·6H₂O, 10⁻⁵ CuCl₂·2H₂O, 0.007 Na₂MoO₄·2H₂O, and 100 NaHCO₃.

The medium was supplemented with 381.90 mg L⁻¹ NH₄Cl (100 mg L⁻¹ N—NH₄), 303.41 mg L⁻¹ NaNO₃ (50 mg L⁻¹ N—NO₃), and 182.18 mg L⁻¹ KH₂PO₄ (41.5 mg L⁻¹ P-PO₄). Details on the working volume of the medium, adjustments to ammonium concentrations, pH levels, and other specific conditions for each assay are provided in Table 1.

**Table 1:** Experimental conditions for microalgae growth and nitrogen removal assays.

Assay	Working Volume (mL)	NH₄-N (mg L⁻¹)	pH	Special Notes
Nitrogen compouns removal assay	1000	100	7.5	Molar ratios mimicking real effluents (N—NH₄: N—NO₃ = 2:1; N:*P* = 8:1).
High-ammonium stress assay	500	2000^a^	7.5	a Medium enriched with additional NH_4_Cl
Acidophile screening	500	100	3.0	Buffered with trisodium citrate
Alkalophile screening	500	100	10.0	Buffered with TRIS base

a final concentration of ammonium chloride (NH₄Cl) in the medium was 7641.4 mg l^-1^.

The initial concentration of free ammonia (FA) in the medium was calculated to be 103.54 mg L⁻¹ at pH 10 (alkaline assay) and 2.15 mg L⁻¹ at pH 7.5 (nitrogen compound removal assay). These values were determined using Eq. (1) as described by [25,26].

(1) $$FA=\frac{\left[NH_{4}-N\right]}{1+\frac{10^{-pH}}{10^{-\left(0.09018+\frac{2729.92}{T\left(K\right)}\right)\;}}}\times \frac{17}{14}$$

#### 2.4.2 General experimental conditions

All assays were conducted in triplicate for each strain under a batch cultivation setup in Erlenmeyer flasks over a 12-day experimental period, with an initial cell density of 1 × 10⁶ cells mL⁻¹. Microalgal cells from each strain in the logarithmic growth phase were harvested from stock cultures by centrifugation at 4000 × *g* for 1 min and resuspended in distilled water prior to inoculation.

The cultures were maintained at a controlled room temperature of 25 °C, with constant aeration and illumination provided by cool white light at an intensity of 100 µmol m⁻² s⁻¹. A 12:12 light/dark photoperiod was employed throughout the experiment. Sampling for analysis was performed every 2 days.

Each experiment included a control group, which consisted of a synthetic medium without algal cells. This control was maintained under the same conditions as the experimental groups.

### 2.5 Analytical methods

#### 2.5.1 Wastewater characterization

Physicochemical analyses of the wastewater samples included pH, conductivity, temperature, dissolved oxygen (DO), biochemical oxygen demand (BOD), nitrogen compounds, and total phosphorus. All analyses followed standard methods prescribed for water and wastewater examination.

#### 2.5.2 Nutrient analysis

For the nitrogen compound removal experiments, samples (5mL) were collected from each treatment and centrifuged at 4000 × *g* for 5 min to separate the microalgae biomass from the culture medium. The concentrations of ammonium (NH₄-N) and nitrate (NO₃-N) in the supernatant were measured using photometetric methods with specific reaction kits. Ammonium nitrogen was quantified using the Ammonium Test, Photometric Method 2.0 (range: 1.50–150 mg/L NH₄-N; 2.6–193 mg/L NH₄⁺, Spectroquant®). Nitrate nitrogen was determined with the Nitrate Test, DMP Method (range: 0.10–25.0 mg/L NO₃-N; 0.4–110.7 mg/L NO₃⁻, Spectroquant®). The calculations for the nutrient removal efficiency were as follows Eq. (2)

(2) $$Nutrient\;Removal\;\%=\frac{C_{i}-C_{f}}{C_{i}}\;\times \;100\;\%$$

Where *C_i_* represents the initial concentration and *C_f_* denotes the final concentration of the respective compound.

#### 2.5.3 Microalgal growth

For the nitrogen compound removal experiments, microalgal growth was monitored by measuring the cell density of the cultures using a Neubauer improved hemocytometer with a 0.1 mm depth (Marienfeld, Germany) under an optical microscope at 40 × magnification (Axiolab, Zeiss, Switzerland). Additionally, biomass accumulation was quantified by determining the dry weight. To achieve this, the pellet obtained from the centrifugation step described previously was resuspended in distilled water, filtered through a 0.45 µm membrane, and dried at 80 °C for 24 h. The dry weight was then measured using a microbalance.

During the exponential growth phase, the specific growth rate was calculated according to Eq. (3)

(3) $$\mu \;\left(d^{-1}\right)=\frac{lnx_{1}-\;lnx_{0}}{t_{1}-\;t_{0}}$$

where X_1_ and X_0_ are the cell concentrations at time t_1_ and t_0_ (end and beginning of exponential growth phase), respectively.

For extreme condition assays, 1 mL culture test samples were subjected to optical density measurements at 686 nm (OD686) using a spectrophotometer. Cell density was calculated using calibration curves determined for each microalga strain. The relationship between biomass dry weight (cells mL⁻¹) and optical density (OD686) was estimated using linear regression models.

### 2.6 Statistical analysis

Microalgae growth and nitrogen nutrient removal were compared using one-way analysis of variance (ANOVA) to assess differences between groups. Data were presented as mean ± standard deviation (SD) for each treatment. Significant differences identified by ANOVA (*p* < 0.05) were further analyzed using multiple pair-wise comparisons with the least significant difference (LSD) test. Statistical analyses were conducted using R software (version 4.2.2), and graphs were developed using pyFOOMB.

## 3 Results

### 3.1 Physicochemical analysis of sampling sites

The sites selected for the bioprospecting of microalgae capable of removing nitrogen compounds were located downstream of discharges from several pig farms with an ammonium concentration gradient ranging from 77.4 to 285.4 mg l^-1^. Two sampling points were established in an oxidation pond collecting pig effluents, with ammonium concentrations of 201.4 mg l^-1^ (OP1) and 285.4 mg l^-1^ (OP2). Two additional sampling points were situated in the La Paz waterfalls, which directly receives effluent from several pig farms: LP1 with an ammonium concentration of 77.4 mg l^-1^ and LP2 with an ammonium concentration of 124.4 mg l^-1^ (Table 2).

**Table 2:** Physicochemical parameters of swine farm wastewater.

		Physicochemical Parameters								
Sampling Points	Sample	pH	CE	Temp	DO	BOD	NO_3—_N	NO_2—_N	NH_4—_N	TP
			uS cm^-1^	°C	mg l^-1^	mg l^-1^	mg l^-1^	mg l^-1^	mg l^-1^	mg l^-1^
“La Paz” Waterfalls	LP1	7.09	132.2	25	8.9	220	43.5	4.3	77.4	200.1
	LP2	7.78	417.9	28	37.6	500	92.3	6.9	124.4	378.2
Oxidation Pond	OP1	8.52	1247.9	25	54.2	1050	274.1	27.4	201.4	467.5
	OP2	8.71	1569.2	25	68.4	2170	351.7	30.1	285.4	541.8

Note: Temp: Temperature; EC: Electrical conductivity; DO: Dissolved oxygen; NO_3—_N: Nitrate; NO_2—_N: Nitrite; NH_4—_N: ammonium nitrogen; TP = Total phosphorus.

### 3.2 Morphological characterization of isolated microalgae strains

**Fig. 1:**
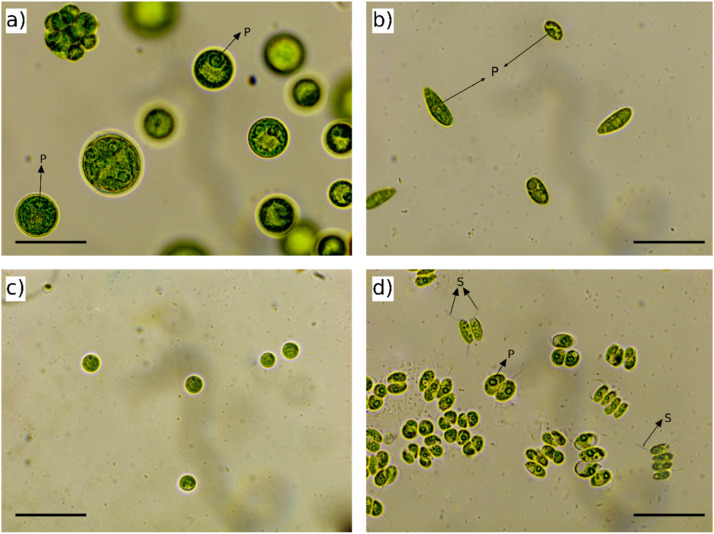
Microalgae isolates observed under 100x magnification using a light microscope. The isolates shown are: (a) LP1, (b) LP2, (c) OP1, and (d) OP2. Pyrenoids (P) and spine-like projections (S) are indicated with arrows for clarity. Scale bar: 25 µm.

One microalgae strain was isolated from each sampling point. The LP1 isolate exhibited spherical cells with a diameter of 8–25 μm, surrounded by a gelatinous envelope. Within this envelope, four to eight cells with one or more visible pyrenoids were observed (Fig._1a). LP2 isolate consisted of ovoid cells (6–15 µm) with a parietal chloroplast and a visible pyrenoid (Fig._1b). OP1 was characterized by small, spherical cells with a diameter of 3–6 µm, which were individually dispersed without any branching (Fig._1c). OP2 comprised green ellipsoidal cells (5–10 µm) organized in coenobium (2–4 cells) and individually. In each of these cells, a central pyrenoid and spine-like projections at the poles were identified (Fig._1d).

### 3.3 Isolates molecular identification

Two genes, 18S and rbcL partial sequences were used for microalgae molecular identifications. The 18S rDNA V1–3 hypervariable region and rbcL partial gene were successfully amplified from DNA samples extracted from the chlorophyte strains. The 18S rDNA sequence of LP1 matched 100% with *Radiococcus polycoccus*, with no other redundant species. LP2 exhibited 100% matches with two different species: *Chlorolobion braunii* and *Ankistrodesmus falcatus*. The OP1 sequence displayed 100% identity with an endosymbiont species from the family Chlorellaceae, as well as with two species of *Chlorella* (*C. sorokiniana* and *C. pyrenoidosa*) and one unidentified species from the genus *Micractinium*. OP2 showed a 95% identity with an unidentified species from the genus *Desmodesmus* (Table 3). rbcL sequences showed only one species-match for each strain, no one with 100 % similarity except for OP2. LP1-rbcL matched the same species as LP1–18S although with a 99,77 % similarity. Strain LP2 showed a 99.62 % similarity with *Chlorolobion braunii*, OP1 exhibited a 97.88 % with *Micractinium singularis* and OP2 displayed 100% identity with *Desmodesmus multivariabilis* (Table 3).

**Table 3:** Identification of the strains isolated from piggery leachate, including the name of the identified species in the GenBank database, percentage of identity and accession number, based on the 18S V1–3 and rbcL marker sequence.

Isolates	18S	%Id	Closest match	rbcL	%Id	Closest match
**LP1**	*Radiococcus polycoccus*	100	AF388378.1	*Radiococcus polycoccus*	99	HM852437.1
**LP2**	*Chlorolobion braunii*	100	KT833587.1	*Chlorolobion braunii*	99	KT355742.1
	*Ankistrodesmus falcatus*	100	X91263.1			
**OP1**	*Chlorella sorokiniana*	100	AB240151.1	*Chlorella* sp.	98	MK295222.1
	*Micractinium* sp.	100	MK235183.1	*Micractinium singularis*	97	MN894287.1
	Chlorellaceae endosymbiont	100	MT040853.1			
	*Auxenochlorella pyrenoidosa*	100	HQ834484.1			
**OP2**	*Desmodesmus* sp.	95	KR061994.1	*Desmodesmus multivariabilis*	100	OK626426.1

**Fig. 2:**
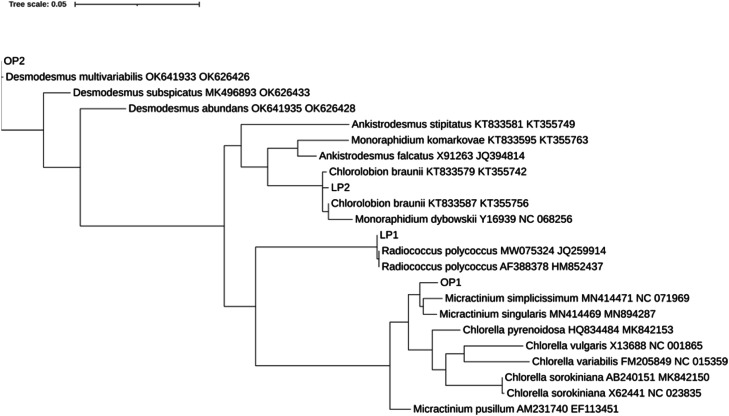
Phylogenetic tree of 18SV1–3 rDNA sequences joined with rbcL sequences from the isolates and GenBank database. The tree was constructed using the maximum-likelihood method with the GTR+*G* + *I* model.

These results indicate successful species-level identification for LP1 (*Radiococcus polycoccus*), LP2 (*Chlorolobion braunii*), and OP2 (*Desmodesmus multivariabilis*). However, OP1 remained ambiguous. To address this, a phylogenetic analysis was performed concatenated 18S and rbcL genes, along with reference sequences from *Micractinium, Chlorella, Ankistrodesmus*, and *Monoraphidium* species (Fig._2). As expected, LP1, LP2, and OP2 clustered closely with their respective species in both rbcL and 18S analyses. OP1, initially presumed to belong to *Chlorella*, grouped with *Micractinium* species instead. Therefore, OP1 was identified as a member of the *Micractinium* genus.

### 3.4 Nitrogen compounds removal-capacity

**Fig. 3:**
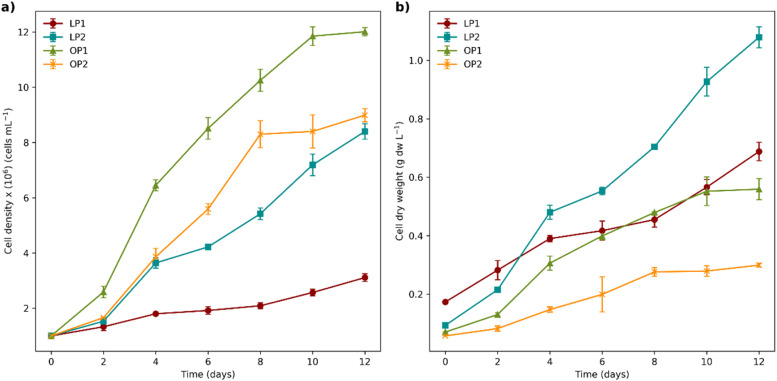
Cell Growth Kinetics. (a) Cell density and (b) Dry weight achieved by each isolate over the treatment time. Data are presented as mean ± SD (*n* = 3 replicate cultures for each strain). Significant differences were observed (*p* < 0.05).

OP1 exhibited the highest cell growth rate, reaching a final cellular density of approximately 1.2 × 10^7^ cells mL^-1^, followed by OP2 (8.99 × 10^6^) and LP2 (8.40 × 10^6^). LP1 had the lowest final cell density, reaching an average of 3.11 × 10^6^ cells mL^-1^ by the end of the trial (Fig._3a). The growth rate calculations (Fig._S1) were performed during the exponential growth phase, which occurred within the first four days. OP1 exhibited the highest growth rate (µ = 0.48 day⁻¹), while LP1 showed the lowest (µ = 0.15 day⁻¹). In contrast, LP2 and OP2 displayed no significant differences in their growth rates during this phase, achieving values of µ = 0.43 day⁻¹ and µ = 0.42 day⁻¹, respectively.

The highest biomass yield was recorded for the LP2 isolate, achieving 1.08 ± 0.04 g cell dry weight per liter (g dw l^-1^), followed by 0.69 ± 0.03 g dw l^-1^ for LP1 and 0.56 ± 0.04 g dw l^-1^ for OP1. The lowest biomass production was observed in OP2 (0.30± 0.01 g dw l^-1^) (Fig._3b).

**Fig. 4:**
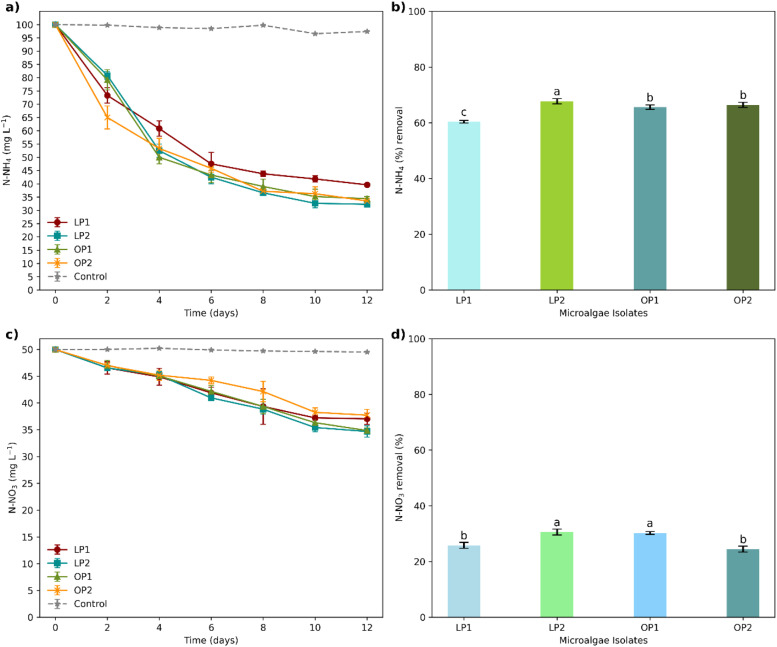
Kinetics and efficiency percentage of N—NH₄ (a, b) and N—NO₃ (c, d) removal by isolates during the assay. Different letters indicate statistically significant differences between groups (*p* < 0.05). Error bars represent mean ± 1 SD (3 replicate cultures for each strain).

The removal of N—NH_4_ varied among the different strains, with statistically significant differences (*p* < 0.05) (Fig._4a). By day twelve, LP2 demonstrated the highest nitrogen removal rate, approximately 67.73%, whereas LP1 exhibited the lowest, at only 60.37%. OP2 and OP1 showed similar nitrogen consumption rates, utilizing around 66.45% and 65.60% of the initial N—NH_4_ concentration, respectively (Fig._4b).

In contrast, the consumption kinetics of N—NO_3_ among strains were comparatively slower than those of N—NH_4_, with significant differences noted only on days 6, 10, and 12 of the trial (Fig._4c). The maximum removal percentages were observed in LP2 (30.58 %), OP1 (30.25 %), LP1 (25.84 %), and OP2 (24.49 %). Statistical analysis revealed no distinction in removal rates between the pairs: OP1/LP2 and LP1/OP2 (Fig._4d).

### 3.5 Strain growth under extreme ammonium and pH conditions

**Fig. 5:**
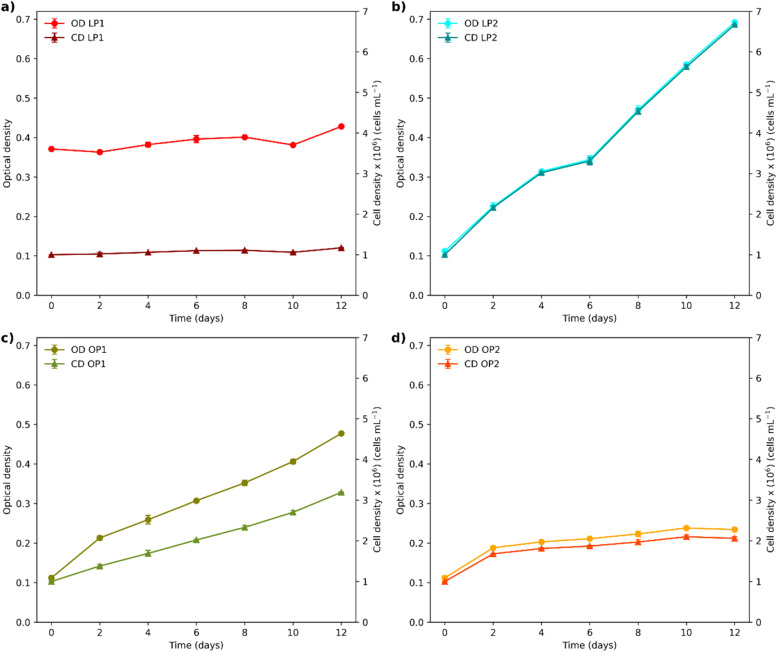
Growth curves of isolates (a) LP1, (b) LP2, (c) OP1, and (d) OP2, based on optical density (OD) and cell density (CD) at 2000 mg L⁻¹ N—NH₄. Data are presented as mean ± SD (*n* = 3). Significant differences were observed (*p* < 0.05).

During the trial at 2000 mg L⁻¹ of N—NH₄, significant differences in growth kinetics were observed among the four strains (*p* < 0.05). LP2 achieved the highest cell density (6.67 × 10⁶ cells mL⁻¹) and optical density (OD) (Fig._5b). OP1 followed, reaching a maximum OD of 0.477 (3.19 × 10⁶ cells mL⁻¹) (Fig._5c). OP2 doubled its cell concentration by day 10 (2.10 × 10⁶ cells mL⁻¹) and remained constant through day 12 (Fig._5d). In contrast, LP1 exhibited the lowest growth (1.17 × 10⁶ cells mL⁻¹) and demonstrated cell aggregation, as indicated by a reduction in optical density (OD) from 0.371 to 0.363 on day 2 and from 0.401 to 0.381 on day 10 (Fig._5a). The pH fluctuated across all cultures, including the control unit. The LP2 culture medium became acidified, reaching a pH of 5 (Fig._S3).

**Fig. 6:**
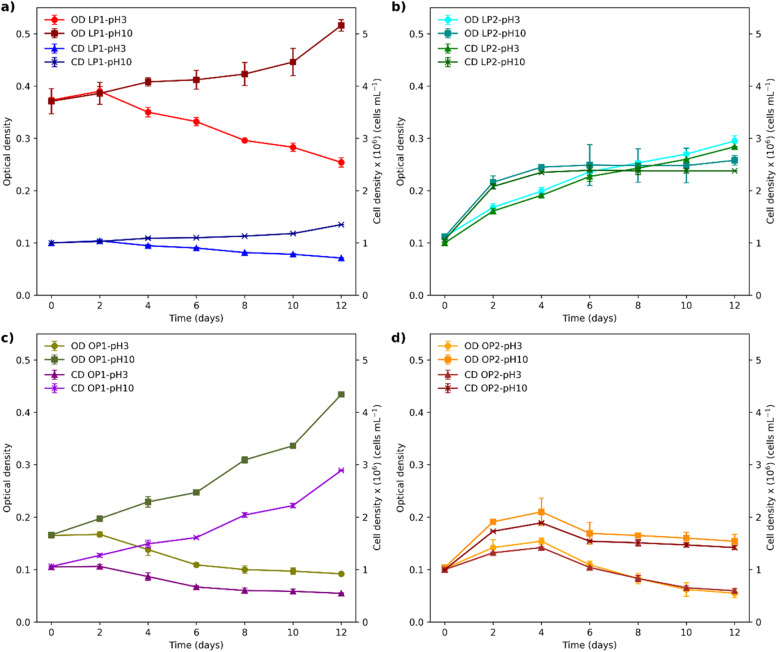
Growth curves of isolates (a) LP1, (b) LP2, (c) OP1, and (d) OP2, based on optical density (OD) and cell density (CD) at pH 3 (acidic) and pH 10 (alkaline). Error bars represent ± SD (*n* = 3). Significant differences were observed (*p* < 0.05).

Under alkaline conditions (pH 10), OP1 showed the highest growth (*p* < 0.05), reaching an optical density of 0.434 and a cell count of 2.89 × 10^6^ cells mL^-1^, followed by LP2 (Fig._6). In contrast, LP1 and OP2 did not achieve a doubling of their cell concentration. At pH 3, only the LP2 strain exhibited growth during the experimental period, showing a significant increase (*p* < 0.05) in cell density, reaching 2.84 × 10⁶ cells mL⁻¹ and an optical density of 0.295. From the second day onward, a phase of cell death was observed in the other strains, with cell concentrations dropping to 10⁵ cells mL⁻¹. At this pH, optical microscopy revealed cell lysis in LP1, OP1, and OP2, while the morphology of LP2 changed, showing more turgid and granulated cells (Fig. S2). No significant differences in pH were observed throughout the test (Fig. S4).

## 4 Discussion

The isolated strains were identified to the species level using a combination of genetic barcode markers, as demonstrated by previous studies [19,27,28]. For LP2, the genera *Chlorolobion* and *Ankistrodesmus,* which have undergone numerous taxonomic reassignments, are considered homotypic synonyms [29] *Monoraphidium* is also considered homotypic with *Chlorolobion braunii* [30]. Thus, despite apparent discrepancies in taxonomic matches, the LP2 strain can be confidently identified as *Chlorolobion braunii.* However, the OP1 strain, morphologically identified as *Chlorella* spp., clustered with *Micractinium* spp., despite lacking typical *Micractinium* morphological features, such as cell aggregations and bristle projections [31,32]. This is consistent with the absence of bristles and the coccoid shape of other *Micractinium* species such as *M. pusillum,* which is often misidentified with *C vulgaris* [33,34]. Nevertheless, OP1 seems to be more related with *M. singularis* and *M. simplicissimum*, also reported as bristle-less subspecies in Antartica [35].

All isolated genera have previously been assessed for nitrogen compound removal, though some have been studied more extensively than others. *Desmodesmus*, for example, showed nitrogen removal rates exceeding 90 % in synthetic media. However, these studies used significantly lower nitrogen concentrations compared to the current experiments [23,[36], [37], [38], [39]]. Although its potential for the removal of nitrogenous compounds has been minimally explored, *C. braunii* has been recognized for its wastewater treatment potential due to its flotation capacity as reported in some biochemical studies [40]. The growth of these strains improves with an increase in nitrogen concentration. When basal concentrations of total nitrogen (TN) are used in BBM medium, *Chlorolobion braunii* tends to exhibit low productivity [41]. With higher nitrogen concentration, there is a significant increase in both productivity and cell density [42]. In the same study, using sodium nitrate as the sole nitrogen source, a maximum cell density of 2.4 × 10⁶ cells was achieved. In contrast, in the present study, with a higher ammonium concentration than nitrate, cell density nearly quadrupled in half the trial time. *Radiococcus nimbatus* has been reported in sewage water treatment [43] and pharmaceutical compound removal [44], but no in-depth kinetic studies or its role in ammonium removal have been conducted. Other studies have reported that *Micractinium* spp. achieved nitrogen removal rates exceeding 90 % within 25 days, with an N:P ratio of 9 [45], and 86% within 8 days at an initial total nitrogen concentration of only 15 mg L⁻¹, reaching biomass levels of up to 0.3 mg L⁻¹ [46].

Previous studies have demonstrated that a ratio of 20:1 N/P is particularly effective for microalgal growth [47,48]. Stumm's empirical formula for microalgae (C₁₀₆H₂₆₃O₁₁₀N₁₆P) suggests that the ideal nitrogen-to-phosphorus (N:P) ratio may range from 7.2:1 to 16:1 [49]. The N:P ratio in pig manure leachates and synthetic medium (8:1) falls within this optimal range. An appropriate nutrient ratio, coupled with the inherent adaptability of native strains to wastewater conditions, can significantly reduce the lag phase in cultures [37]. This is consistent with the growth patterns observed in the isolated strains.

The assimilation rates and removal percentages for N—NH₄ were significantly higher than those for N—NO₃. This is because ammonium consumption requires less energy, as it does not need an electron donor for reduction, unlike nitrate-N [50]. These findings align with the Gompertz model, which explains an initial delay in substrate consumption when two different nitrogen sources are available [23]. This delay was evident in the observed 4-day lag in N—NO₃ uptake, during which all strains achieved over 40% removal of ammonium nitrogen.

In this study, all isolates achieved over 60 % N—NH_4_ removal within 12 days, starting from a concentration of 100 mg l^-1^. These findings emphasize the potential of these strains for treating pig effluents, even at high nitrogen concentrations. This is particularly significant because many commercial strains exhibit toxicity when exposed to ammonium concentrations above 20 mg l^-1^, which impairs growth and results in suboptimal removal rates [36,51].

Studies have suggested that strains can be classified as ammonium-tolerant if they survive concentrations exceeding 200 mg L⁻¹ of N—NH₄ [52]. However, it has been demonstrated in previous research that ammonium concentrations above 100 mg L⁻¹ can inhibit photosynthesis in some microalgal species [53,54]. Therefore, identifying microalgae species with exceptional resistance to NH_4_^+^ toxicity is crucial for developing effective effluent treatments. In this work, the LP2 strain demonstrated remarkable tolerance, growing at ammonium concentrations as high as 2000 mg L⁻¹, with only a 20% reduction in cell growth compared to growth at 100 mg L⁻¹. This observation aligns with findings from other studies suggesting that *Chlorolobion braunii* strains exhibit superior productivity in nitrogen-rich media, particularly under high nitrogen conditions, compared to *Micractinium* strains [55]. To date, no studies have reported the growth performance of *Chlorolobion* species at ammonium concentrations as elevated as 2000 mg L⁻¹.

High ammonium concentrations also disrupt proton balance both within cells and in the surrounding medium, reducing growth productivity even in acid-tolerant strains [9,56,57]. In this context, pH plays a critical role in regulating microalgal growth by maintaining the NH₃/NH₄⁺ equilibrium and nitrogen availability. Extreme pH and elevated ammonium levels can inhibit carbohydrate synthesis and impair enzymes involved in photosynthesis [58], ultimately decreasing the growth rate [[54], [59]].

Microalgae have specific pH ranges for optimal growth, but different species exhibit varying levels of adaptability to these conditions [9]. While most microalgae prefer neutral pH, several species have been reported to thrive under acidic (pH < 5) or alkaline (pH > 9) conditions [60]. Some algal species can tolerate both acidic and basic environments [61]. Particularly, LP2 growth was not inhibited under acidic and alkaline stress, marking the first report of *Chlorolobion* growth under extremophile pH conditions. Under pH 3, LP2 exhibited reduced growth, smaller cell size, and increased granularity, consistent with stress adaptation mechanisms described for other extremophilic microalgae [[61], [62], [63], [64], [65], [66]].

The growth inhibition of the other strains was evident at acidic pH, but they were alkaline-tolerant, improving their growth as it approached neutrality. OP1 was the most alkaline-tolerant strain. *Micractinium* sp. has been reported to grow in alkaline conditions up to pH 10 [67]. However, at extremely acidic pH levels chlorophyll *a* degradation and cell lysis [68], as observed at pH 3 in OP1–2 and LP1. *Desmodesmus* spp*.* have been reported to grow at pH levels of 3 to 3.5 [69,70]. However, Scenedesmaceae species prefer neutral conditions and can tolerate alkaline environments [69], which is consistent with the current findings.

Alkaline conditions at pH 10 have the potential to shift the ammonium equilibrium toward free ammonia (FA), a compound toxic to aquatic biota [26]. The effect of alkaline pH on microalgal growth observed in this research cannot be solely attributed to the pH *per se* but is rather influenced by the production of free ammonia induced by high pH levels. The generation of FA is highly dependent on pH and the total ammoniacal nitrogen (TAN) concentration and has been recognized as a source of toxicity to microalgal cells [[70], [71], [72]]. It has been proposed that the FA/TAN ratio can increase up to tenfold with every unit increase in pH [26], pH indirectly impacts microalgal growth due to the associated production of FA.

When comparing the effects of different pH values at a constant TAN concentration on microalgal growth, the optimal pH range for microalgal growth and productivity was determined to be between 6.5 and 7.5 [26], as established in the first experiment of this study. As pH increased, growth gradually declined, except at low TAN concentrations (50 mg l^-1^ or less) [73]. At higher ammonium concentrations (as in the present study), growth reduction due to the joint effect of pH and FA concentration could exceed 50% [74]. These findings align with the observed results, where at a pH of 7.5, as evaluated in the nitrogen compound removal experiment, the FA concentration was 2.15 mg l^-1^ under an initial ammonium concentration of 100 mg l^-1^, where microalgal growth was 1.25- to 5-fold higher.

The ability to tolerate FA toxicity is known to be species-specific and has been associated with strains found in high-strength wastewaters in natural environments [75,76]. OP1 and LP2, presented notable tolerance by surviving an initial FA concentration of 103.54 mg *L*^−1^. This concentration matches the levels tolerated by other strains that have been cataloged as highly resistant in literature, such as *Chlorella* spp. [26,72,74,77,78]. This ability could provide a cultivation advantage. By increasing the pH and maintaining FA concentrations within a range of 40–50 mg l^-1^, FA could act as a growth inhibitor for potential contaminants in the culture, such as rotifers and water fleas [79]. This characteristic highlights the isolates' potential applicability in controlled cultivation systems with selective growth. Therefore, it is recommended for future studies to evaluate the joint (bifactorial) and independent effects of FA and pH, as well as inhibitory kinetics models, to better understand FA-induced inhibition during microalgal growth.

## 5 Conclusion

This study highlights the potential of indigenous microalgae for optimizing piggery wastewater treatment. The isolates *Radiococcus polycoccus, Chlorolobion braunii, Micractinium* sp., and *Desmodesmus multivariabilis* demonstrated a strong capacity for nitrogen compound removal from synthetic effluents, showing a clear preference for ammonium over nitrate assimilation. *Chlorolobion braunii* exhibited good adaptability, maintaining growth at 2000 mg L⁻¹ of N—NH₄ and tolerating both acidic and alkaline conditions. These traits underscore its promise for sustainable biotechnological applications. The results reveal variability in cell growth under different culture conditions, highlighting the importance of comprehensive strain characterization. This study also presents the first kinetic characterization of *Radiococcus polycoccus*. Future research should focus on elucidating the biochemical pathways and genetic mechanisms underlying these adaptive traits to enhance the efficiency and scalability of microalgae-based bioremediation technologies.

## CRediT authorship contribution statement

**Karla Flores-Zambrano:** Conceptualization, Formal analysis, Investigation, Methodology, Writing – original draft, Writing – review & editing. **Wilson Tapia:** Methodology, Conceptualization, Validation, Funding acquisition. **Pablo Castillejo:** Conceptualization, Resources, Writing – review & editing.

## Declaration of competing interest

The authors declare that they have no known competing financial interests or personal relationships that could have appeared to influence the work reported in this paper. All aspects of this study, including its design, data collection, analysis, and interpretation, are the sole responsibility of the authors and have been conducted independently without any external influence.

## Appendix Supplementary materials

Image, application 1
