# Supplementary material for: Microalgae strains isolated from piggery wastewater in Ecuador: Effective nitrogen compound removal and growth potential in extremophile conditions
Source: Biotechnol Rep (Amst). 2025 Feb 10;45:e00883. doi: 10.1016/j.btre.2025.e00883 (PMC11869989; doi:10.1016/j.btre.2025.e00883)
Supplement: Supplementary file 1 [file mmc1.docx]

Microalgae Strains Isolated from Piggery Wastewater in Ecuador: Effective Nitrogen Compound Removal and Growth Potential in Extremophile Conditions

Karla Flores-Zambrano, Wilson Tapia, Pablo Castillejo

SUPPLEMENTAL DATA


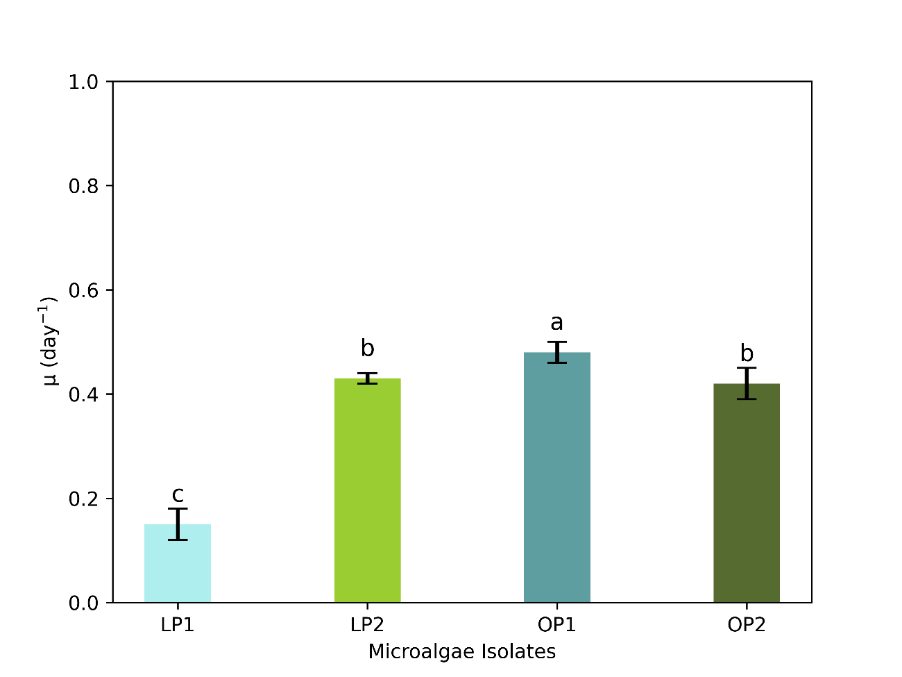


Figure S1. Specific growth rate (µ day⁻¹) based on cell density measurements. Different letters indicate statistically significant differences between groups (p < 0.05). Error bars represent the mean ± 1 standard deviation (SD) from three replicate cultures for each strain.


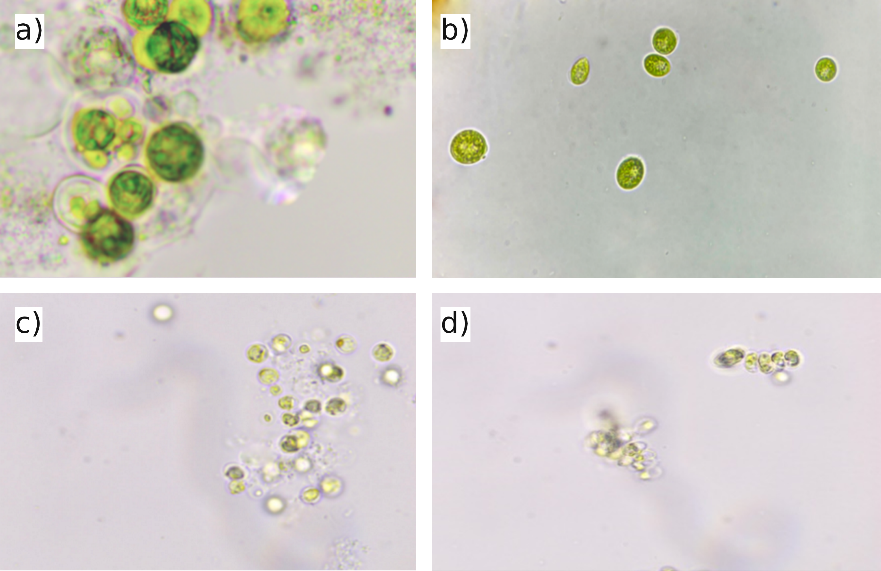


Figure S2. Microalgae isolates observed under 100× magnification using a light microscope during the pH 3 assay. (a) LP1, (b) LP2, (c) OP1, (d) OP2. Morphological changes in LP2 cells include increased turgidity and the presence of internal granules.


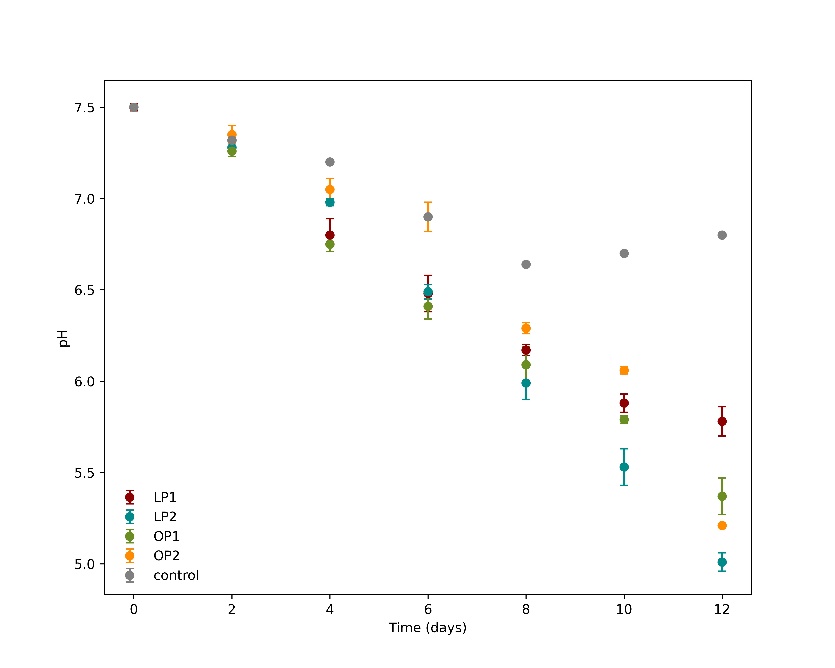


Figure S3. Changes of pH in the culture medium of LP1 (red circle), LP2 (cyan-blue circle), OP1 (green circle), OP2 (yellow circle) and Control (gray circle) during the experiment at 2000 mg L ^-1^ of ammonium concentration in media. Error bars represent mean ± 1 SD (3 replicate cultures for each strain).


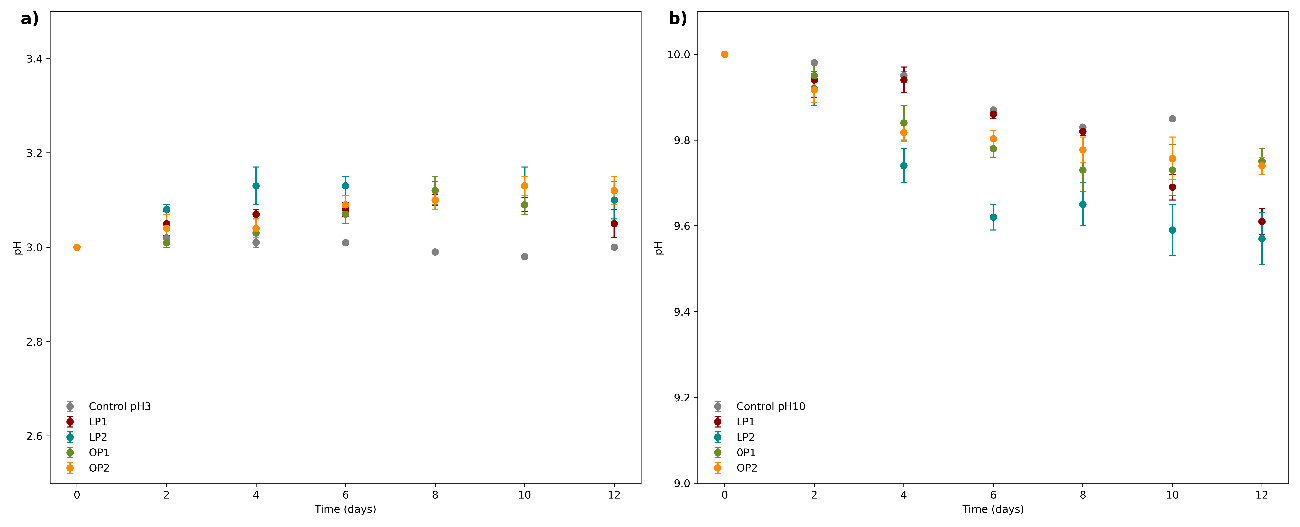


Figure S4. Changes in pH of the culture medium for strains LP1 (red circles), LP2 (cyan-blue circles), OP1 (green circles), OP2 (yellow circles), and the control (gray circles) during the experiment. Panel (a) shows pH variations at an initial pH of 3, while panel (b) shows pH variations at an initial pH of 10, using the same color scheme for both conditions. Error bars represent the mean ± 1 standard deviation (SD) based on three replicate cultures for each strain.
